# PIMIDES I: a pilot study to assess the feasibility of patient-controlled neurostimulation with the EASEE® system to treat medically refractory focal epilepsy

**DOI:** 10.1186/s42466-020-00061-5

**Published:** 2020-06-02

**Authors:** Kristina Kravalis, Andreas Schulze-Bonhage

**Affiliations:** Epilepsy Center, University Hospital Freiburg, Breisacher Str. 64, 79106 Freiburg, Germany

**Keywords:** Medical refractory epilepsy, Neurostimulation (NS), Responsive focus-stimulation

## Abstract

The study design of PIMIDES, a trial based on patient-individualized transcranial electric neurostimulation of epileptic foci, is reported. Inclusion criteria include a predominant epileptic focus and pharmacoresistance to two antiepileptic drug treatments. The study is prospective, unblinded, and serves to assess the safety of subgaleal implantation and transcranial stimulation.

## Introduction

Non-pharmacological treatment of epilepsy is increasingly recognized as an option to improve seizure control and quality of life of epilepsy patients. In particular, pharmacoresistant patients who are no good candidates for epilepsy surgery can profit from extracranial peripheral or intracranial stimulation (deep brain stimulation of the thalami and responsive focus stimulation).

We here report extracranial, subgaleal patient-controlled stimulation of the epileptic focus as a procedure with low invasiveness targeting the epileptogenic brain area. The implantable device EASEE® System was specially developed for subgaleal NS and modified for a patient-controlled stimulation for the purpose of this trial. It is expected to gain knowledge on the feasibility and usefulness of a closed-loop transcranial stimulation system as developed in the BMBF granted PIMIDES project.

## Methods

### Aim of the trail

Primary: to demonstrate the safety and performance of patient- controlled NS with the EASEE® System. Secondary: encompass treatment effects on seizure frequency, severity, duration, and symptoms, also effects on interictal EEG discharges, on cognition, patient mood and quality live.

### Study description and design

PIMIDES is a prospective, interventional, unblinded, multicenter study designed to collect data on 18 subjects implanted with the EASEE® System. The total duration of the study protocol per subject is 17 months, including 1 month of baseline monitoring, 1 month of post-implantation recovery, 3 months of safety evaluation period and 12 months of follow- up to assess efficacy.

### Arm and interventions

**Fig. 1:**
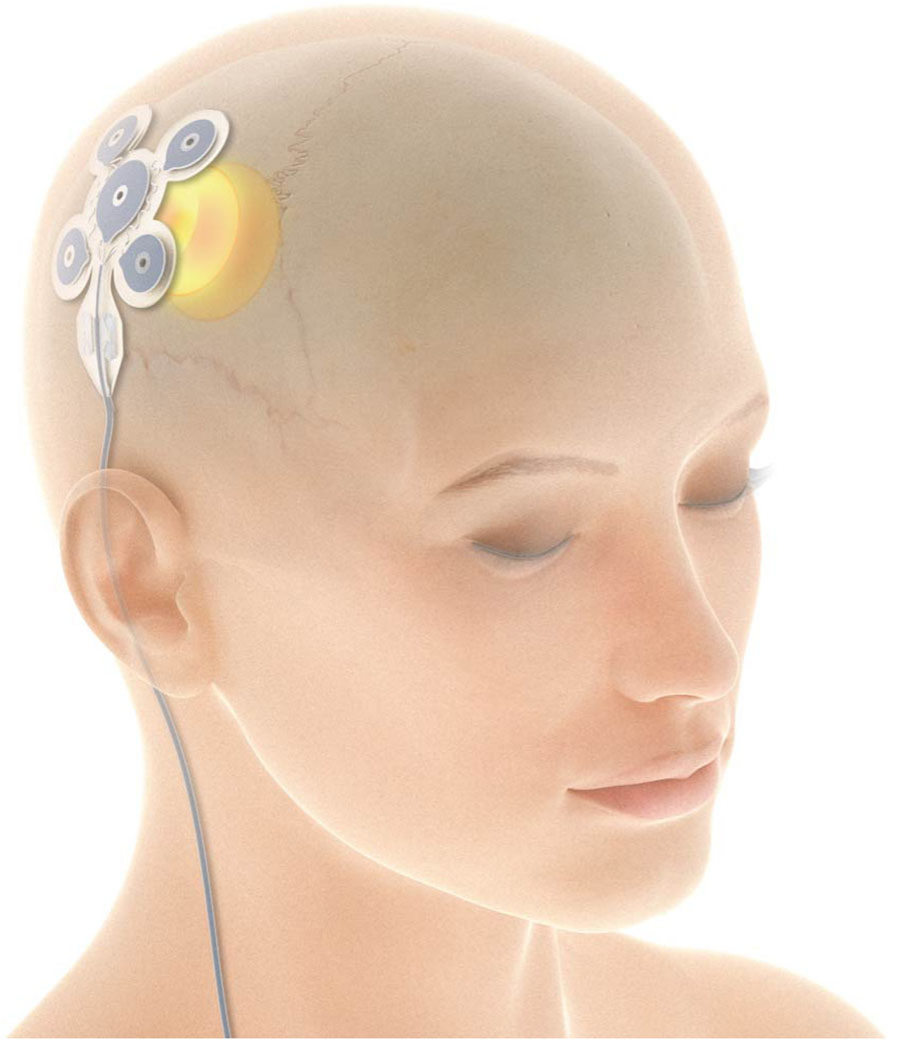
The transcranial focusstimulation is performed through the electrode, which is placed under the skin on the cranium, in the specific place over the predetermined epileptic focus

This is a single-arm trial in which all patients will receive implantation and stimulation treatment. The implantation is performed by a neurosurgeon approximately 1 month after baseline monitoring. The EASEE® LEAD will be fixed on the skull surface after removal of the periosteum. The central electrode is placed in the specific place over the pre-determined epileptic focus and connected subcutaneously to the pulse generator EASEE Power placed subcutaneously on the trunk (Fig. 1).

After 1 month of recovery after the surgical intervention, the stimulation parameters can be configured, and the device will be turned on. Treatment occurs in asymmetric intermittent fashion stimulation by three different modes. The ultra-low frequency stimulation mode with daily burst pulses (single pulse duration 20 ms, burst duration of 20 min) is used for long term neuromodulation. The continuous alternating current high- frequency (100 Hz) stimulation mode is carried out in bursts (single pulse duration 160 μs, burst duration of 500 ms, every 2 min). The patient triggered bolus stimulation is carried out with 160 μs pulse width, 100 Hz bursts applied for 10 to 60 s, once requested by the patient using the external EASEE® Access handheld device.

Stimulation intensity is adapted individually patient in a range from 0.1–4 mA.

### Outcome measures

Primary outcome measures are perioperative and chronic safety. Secondary outcomes encompass treatment effects on seizure frequency, severity, duration and symptoms, effects on interictal EEG discharges, on cognition, mood and quality of life.

### Eligibility

Inclusion criteria:

1. Patients with a clinical diagnosis of focal seizures or focal to bilateral tonic-clonic seizures.
2. Patients with a diagnosis of lateral temporal lobe or extra- temporal lobe epilepsy.
3. Patients with a predominant epileptic focus, which can be clearly identified as the site of implantation for the electrode based on EEG and clinical presentation.
4. Patients who are able to initiate a stimulation bolus during their seizure.
5. Patients, after respective surgery, to treat epilepsy, who have clearly identifiable epileptic focus and a preserved neocortex in the region of implantation.
6. Patients who have failed treatment with a minimum of two anti- seizure medications (used in appropriate doses).
7. Patients having seizures, which are district, stereotypical events and can be reliably counted, in the opinion of the investigator, by the patient or caregiver and recorded in a seizure dairy.
8. Patients having an anticipated average of 3–200 partial- onset seizures (focal to bilateral tonic-clonic seizures) during the baseline period.
9. Patients taking a constant dose of antiepileptic medication(s) over the most recent 28- day period to the baseline period (use the medication for acute treatment of seizures is allowed)
10. Patients between the ages 18 and 75 years.
11. Patients able and willing to provide appropriate consent prior to study procedures.
12. Patients able to complete regular office appointments per the protocol requirements, including behavioral (mood) surveys and neuropsychological testing.
13. Patients willing to be implanted with the EASEE® System as a treatment for his/her seizures.

Exclusion criteria:

1. Patients with a diagnosis of mesial temporal lobe epilepsy.
2. Patients with a previous diagnosis of psychogenic or non-epileptic seizures, which are semiologically non-distinguishable from epileptic seizures.
3. Patient with a diagnosis of primary generalized seizures.
4. Patients, if after resective surgery, with non- preserved neocortex in the region of implantation.
5. Patient with unprovoked status epilepticus in the preceding 6 months prior to enrolment.
6. Patients with a clinically significant or unstable medical condition (including cardiac conditions, alcohol and/or drug abuse) or a progressive central nervous system disease.
7. Patients with a diagnosis of active psychosis, major depression, or suicidal ideation in the preceding year (excluding postictal psychosis).
8. Females who are pregnant or have a pregnancy wish in the next 2 years.
9. Patients enrolled in a therapeutic investigation drug or device trail.
10. Patients who are anatomically not eligible for EASEE® System implant in the option of the Investigator.
11. Patients with an implanted electronic medical device that delivers electrical energy to the body (e.g. DBS, cardiac pacemaker or defibrillator) except for an existing VNS device that can be reliably switched off for the duration of the trial.
12. Patients requiring scheduled MRIs during the study phase.
13. Patients who are unable to properly operate the EASEE® Access handheld device. 

## Data Availability

Not applicable.

## Abbreviations

DBS: Deep brain stimulation
EASEE: Epicranial application of Stimulation Electrodes for Epilepsy
EEG: Electroencephalography
MRI: Magnetic resonance imaging
NS: Neurostimulation
PIMIDES: Patient-individualized modulation and intervention by epicranial stimulation
VNS: Vagus nerve stimulation
ms: Milliseconds
μs: Microseconds
mA: Milli- Ampere
Hz: Herz

